# Supplementary material for: Bayesian refinement of protein structures and ensembles against SAXS data using molecular dynamics
Source: PLoS Comput Biol. 2017 Oct 18;13(10):e1005800. doi: 10.1371/journal.pcbi.1005800 (PMC5662244; doi:10.1371/journal.pcbi.1005800)
Supplement: S1 Archive — (BZ2) [file pcbi.1005800.s011.tar.bz2 › gromacs_4.62_bayesian_saxs_refi/share/html/online/g_saltbr.html]

g\_saltbr


| g\_saltbr Main Table of Contents |

**VERSION 4.6  
Sat 19 Jan 2013**

---

### Description

g\_saltbr plots the distance between all combination of charged groups
as a function of time. The groups are combined in different ways.
A minimum distance can be given (i.e. a cut-off), such that groups
that are never closer than that distance will not be plotted.

Output will be in a number of fixed filenames, min-min.xvg, plus-min.xvg
and plus-plus.xvg, or files for every individual ion pair if the -sep
option is selected. In this case, files are named as sb-(Resname)(Resnr)-(Atomnr).
There may be **many** such files.

### Files

| option | filename | type | description |
| --- | --- | --- | --- |
| **-f** | traj.xtc | Input | Trajectory: xtc trr trj gro g96 pdb cpt |
| **-s** | topol.tpr | Input | Run input file: tpr tpb tpa |

### Other options

| option | type | default | description |
| --- | --- | --- | --- |
| **-[no]h** | bool | no | Print help info and quit || **-[no]version** | bool | no | Print version info and quit || **-nice** | int | 19 | Set the nicelevel || **-b** | time | 0 | First frame (ps) to read from trajectory || **-e** | time | 0 | Last frame (ps) to read from trajectory || **-dt** | time | 0 | Only use frame when t MOD dt = first time (ps) || **-t** | real | 1000 | Groups that are never closer than this distance are not plotted || **-[no]sep** | bool | no | Use separate files for each interaction (may be MANY) |

---

http://www.gromacs.org  
gromacs@gromacs.org
